# Supplementary material for: PRPF8-associated retinitis pigmentosa variant induces human neural retina-autonomous photoreceptor defects
Source: Sci Rep. 2026 Feb 23;16:10264. doi: 10.1038/s41598-026-40376-y (PMC13031808; doi:10.1038/s41598-026-40376-y)
Supplement: Supplementary file 1 — Supplementary Information 1. [file 41598_2026_40376_MOESM1_ESM.pdf]

Figure S1

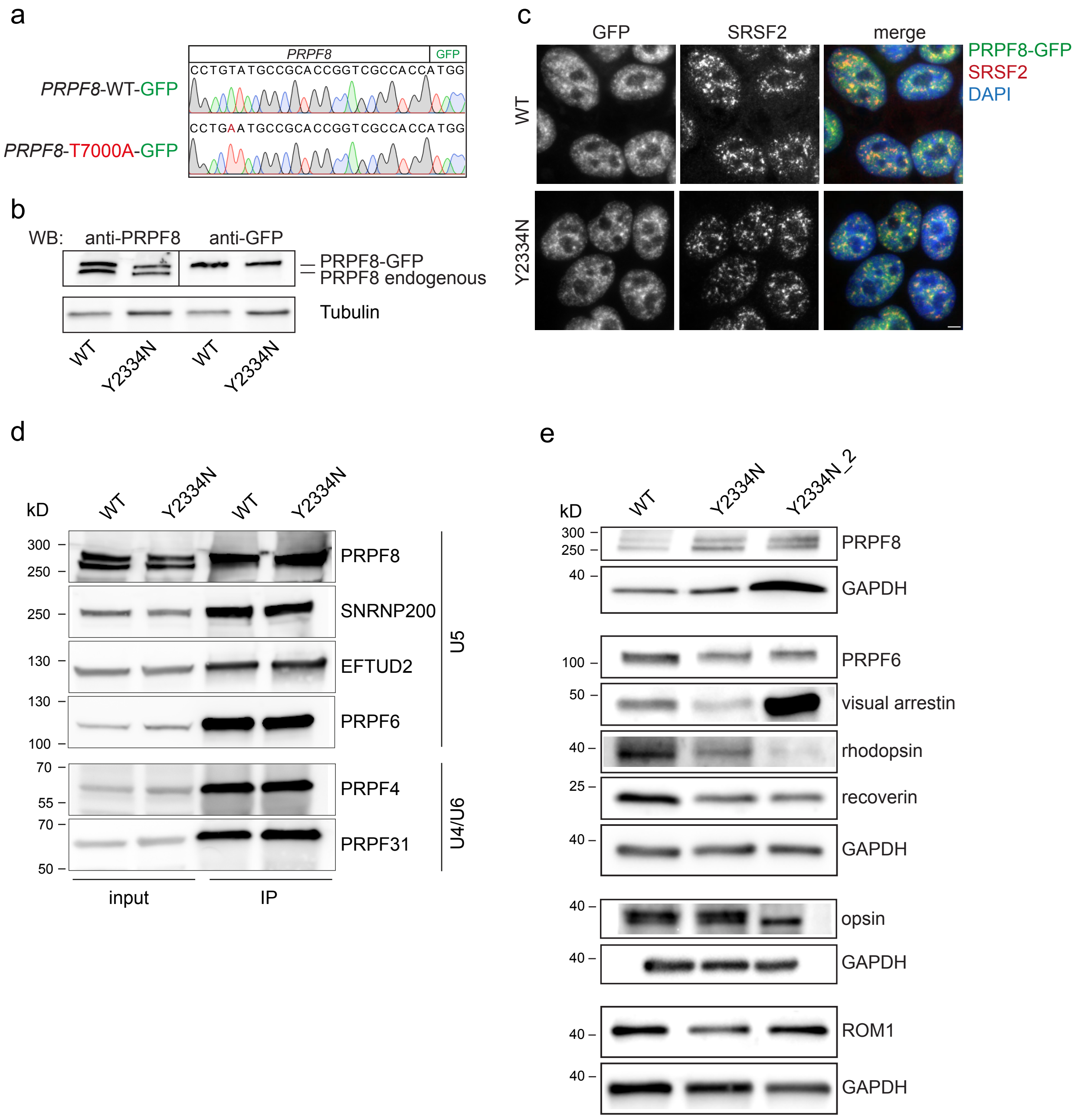

a
PRPF8
GFP
PRPF8-WT-GFP
CCTGTATGCCGCACCGGTCGCCACCATGG
PRPF8-T7000A-GFP
CCTGAATGCCGCACCGGTCGCCACCATGG
b
WB: anti-PRPF8 anti-GFP
PRPF8-GFP
PRPF8 endogenous
Tubulin
WT
Y2334N
WT
Y2334N
c
GFP
SRSF2
merge
PRPF8-GFP
SRSF2
DAPI
WT
Y2334N
d
kD
WT
Y2334N
WT
Y2334N
300
250
PRPF8
250
SNRNP200
130
EFTUD2
130
100
PRPF6
U5
70
55
PRPF4
70
50
PRPF31
U4/U6
input
IP
e
kD
WT
Y2334N
Y2334N_2
300
250
PRPF8
40
GAPDH
100
PRPF6
50
visual arrestin
40
rhodopsin
25
recoverin
40
GAPDH
40
opsin
40
GAPDH
40
ROM1
40
GAPDH

Figure S2

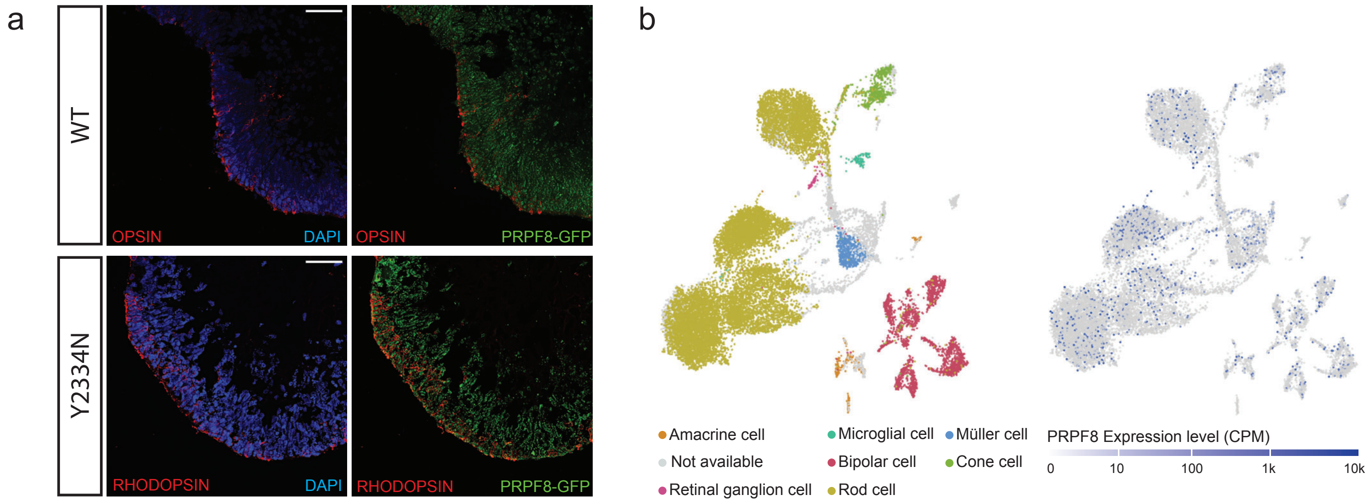
a
WT
Y2334N
OPSIN
DAPI
PRPF8-GFP
RHODOPSIN
b
Amacrine cell
Not available
Retinal ganglion cell
Microglial cell
Bipolar cell
Rod cell
Müller cell
Cone cell
PRPF8 Expression level (CPM)
0
10
100
1k
10k

Figure S3

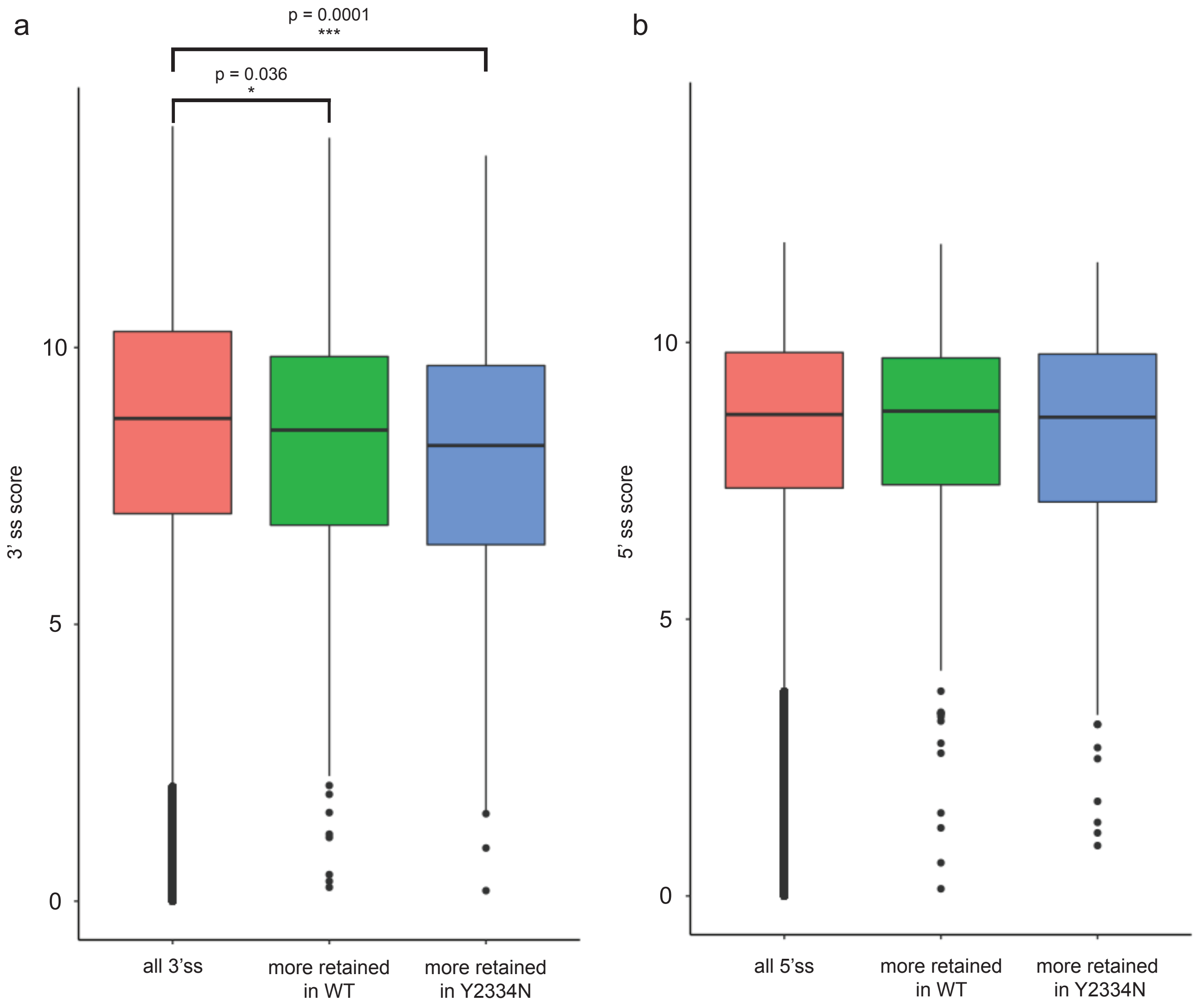

a
p = 0.0001
***
p = 0.036
*
3' ss score
10
5
0
all 3'ss
more retained in WT
more retained in Y2334N
b
5' ss score
10
5
0
all 5'ss
more retained in WT
more retained in Y2334N

Supplementary figure legend

Figure S1. Characterization of human induced pluripotent stem cells (hiPSC) expressing PRPF8-Y2334N-GFP and PRPF8-WT-GFP.

a) Sequence of the GFP-tagged PRPF8 C-terminus of PRPF8-WT and PRPF8-Y2334N hiPSC clones. The c.T7000A substitution in the *PRPF8* gene is highlighted in red. b) Western blot analysis confirming monoallelic editing of the *PRPF8* gene. c) Immunofluorescence analysis reveals that both WT and Y2334N mutant localize to the nucleoplasm and are enriched within splicing speckles as indicated by colocalization with SRSF2. Scale bar = 5 µm. d) Immunoprecipitation (IP) of PRPF8-GFP by anti-GFP antibodies and detection of spliceosomal proteins by western blotting. Inputs contained 2% of the total lysate. e) Western blot analysis of tri-snRNP proteins PRPF8 and PRPF6 and various retinal markers in lysates from PRPF8-WT organoids and PRPF8-Y2334N retinal organoids differentiated from two different PRPF8-Y2334N clones (Y2334N and Y2334N_2).

Figure S2. PRPF8-GFP and PRPF8-Y2334N-GFP are uniformly expressed throughout the neural retina in 170-day-old retinal organoids.

a) Immunofluorescence of indicated retinal proteins and detection of PRPF8-GFP in retinal organoids. b) Analysis of publicly available single-cell RNA sequencing data from the Single Cell Expression Atlas (E-MTAB-7316) reveals uniform expression of PRPF8 in the human neural retina.

Figure S3. The splice site strength of differentially retained introns is reduced.

a) Comparison of the 3′ splice site strength of differentially retained introns in 170-day-old retinal organoids reveals significantly lower strength for introns more retained in Y2334N retinal organoids than the overall human genome average. b) Comparison of the 5′ splice site strength of differentially retained introns reveals no significant difference in strength of differentially retained introns compared to the overall genome average. All 3′/5′ ss refers to the total number of splice sites annotated in the GENCODE (release 47) database. Statistical significance was determined using a Wilcoxon rank sum test with continuity correction (* = $p < 0.05$, *** = $p < 0.001$).
