# Supplementary material for: PRPF8-associated retinitis pigmentosa variant induces human neural retina-autonomous photoreceptor defects
Source: Sci Rep. 2026 Feb 23;16:10264. doi: 10.1038/s41598-026-40376-y (PMC13031808; doi:10.1038/s41598-026-40376-y)

Original data for figure 1C, bottom panel

The rectangle marks the cropped area displayed in figure 1C

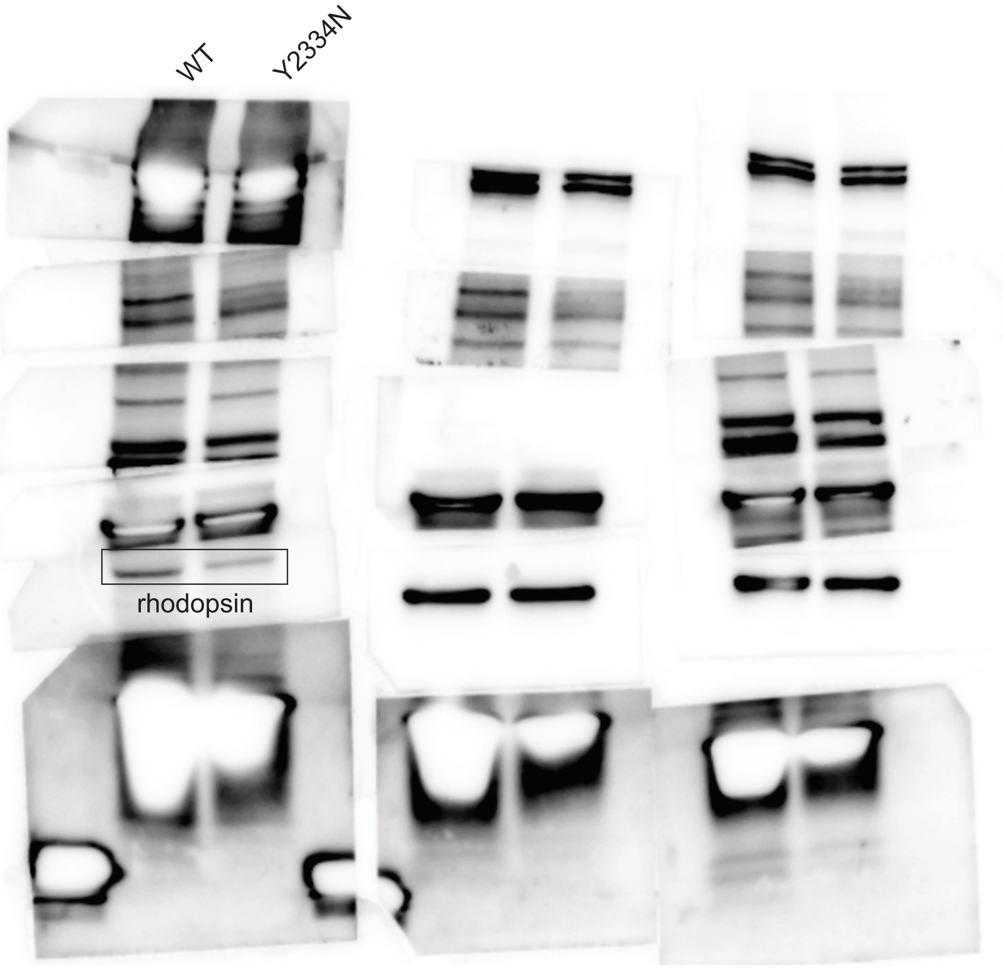

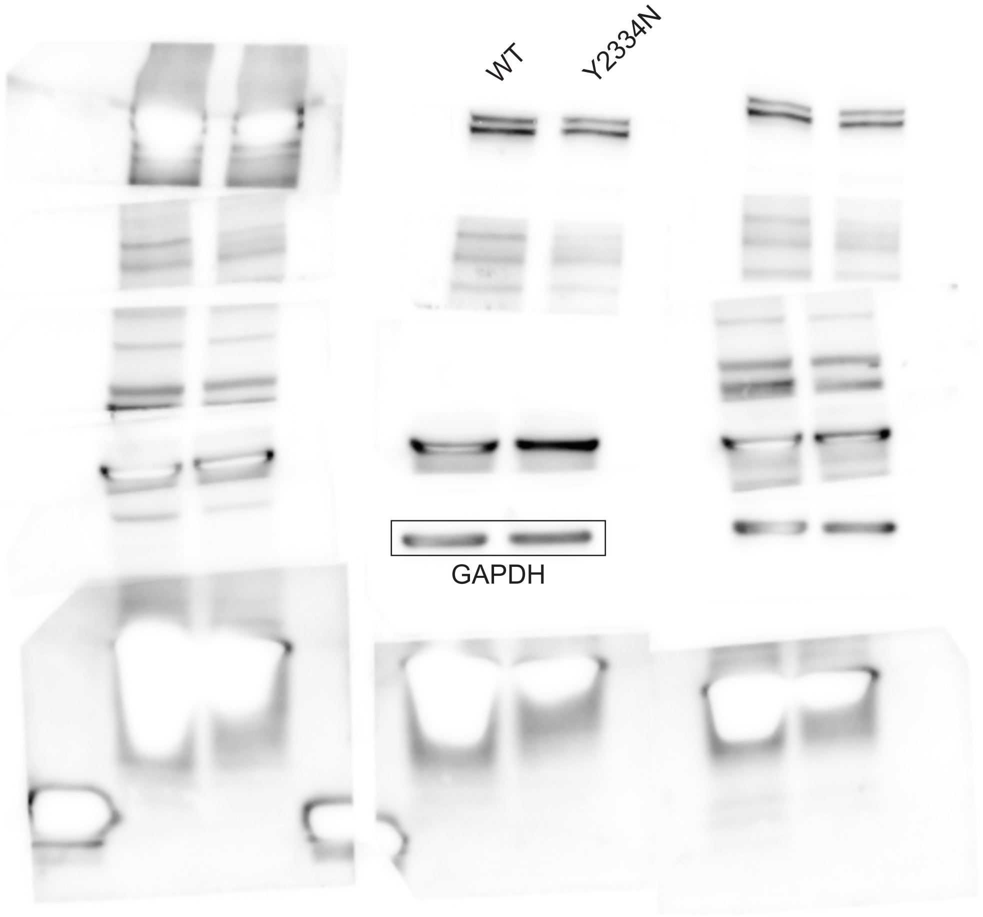

Original data for figure 1C, top panel

The rectangle marks the cropped area displayed in figure 1C

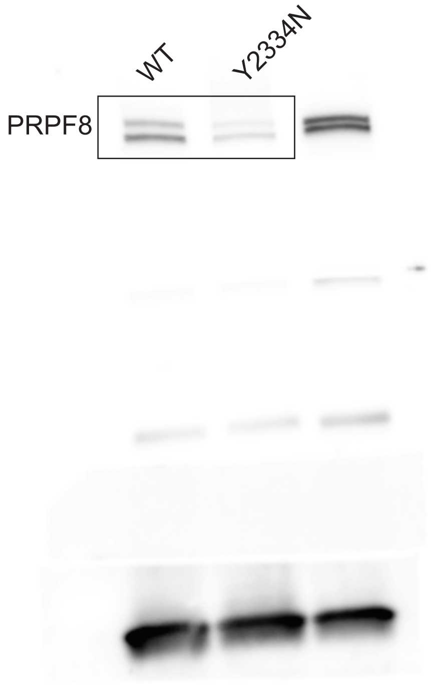

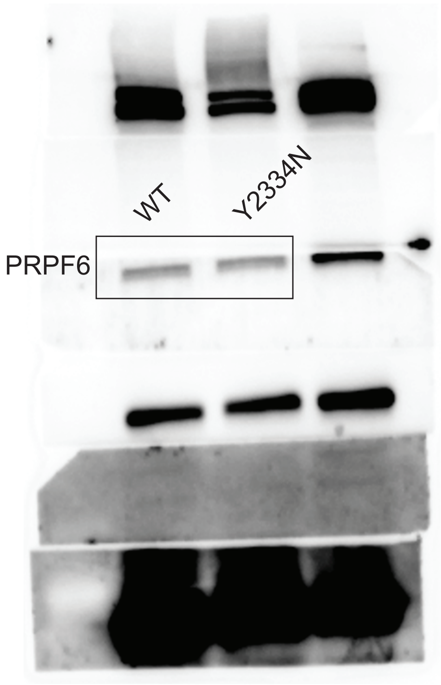

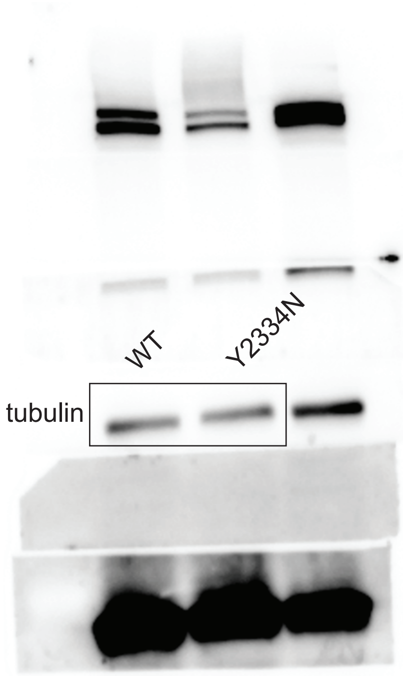

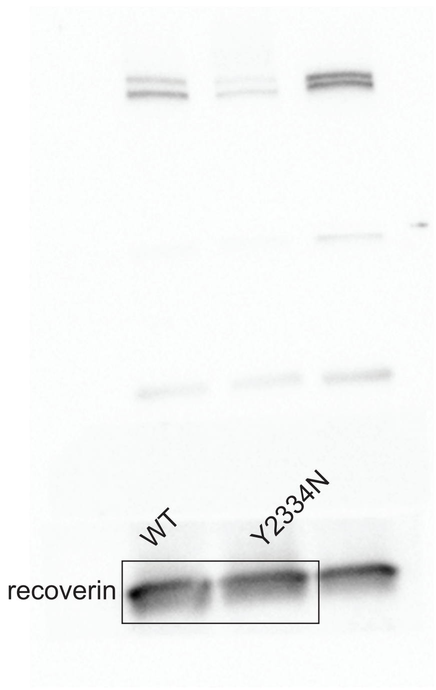

Original data for figure 1d, opsin

The rectangle marks the analyzed data represented by the data point in figure 1d

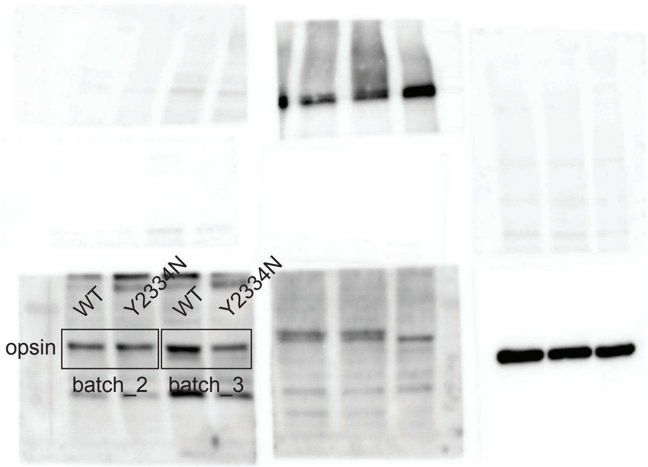

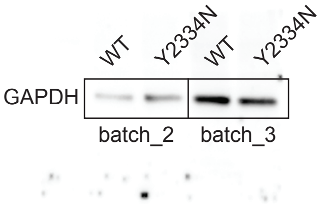

Original data for figure 1d, PRPF6

The rectangle marks the analyzed data represented by the data point in figure 1d

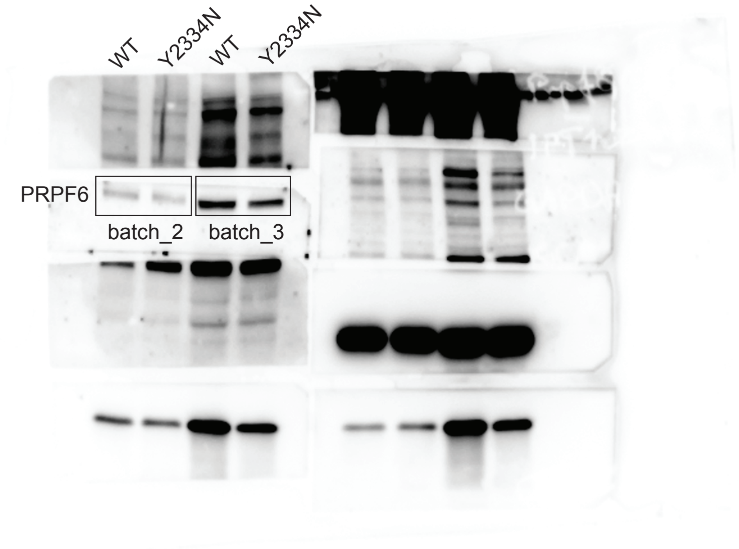

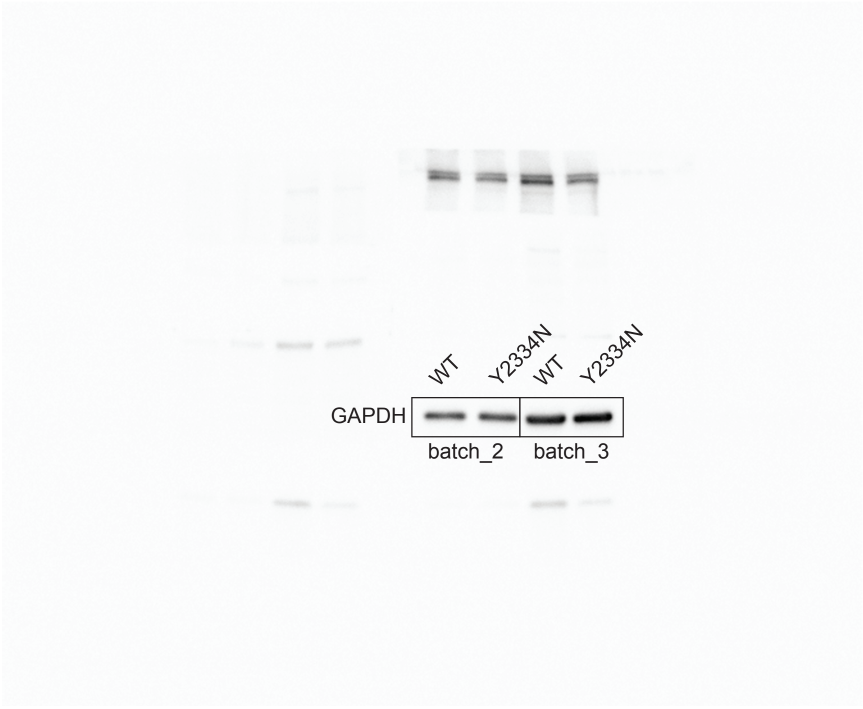

Original data for figure 1d, PRPF8

The rectangle marks the analyzed data represented by the data point in figure 1d

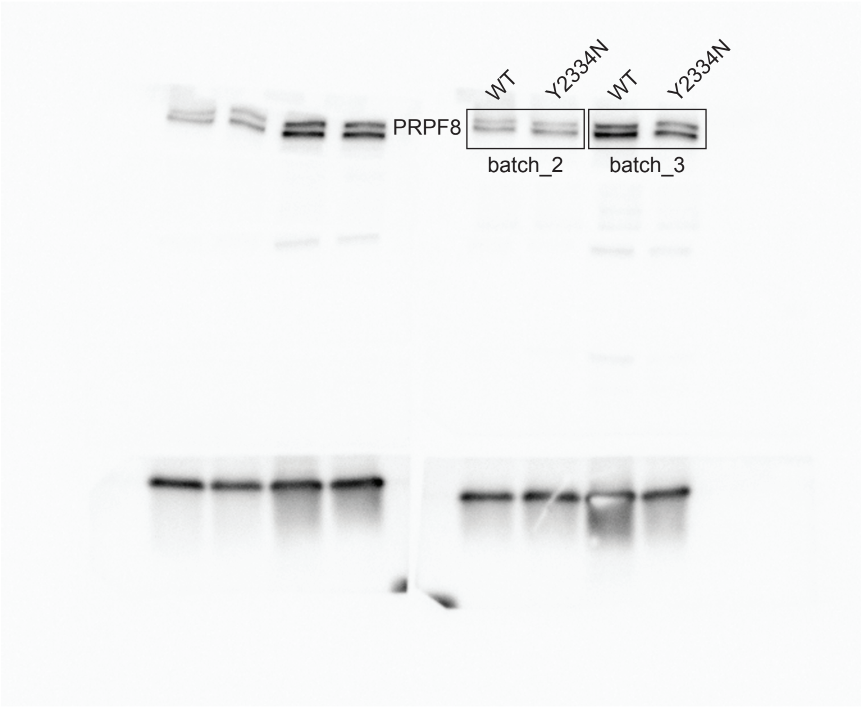

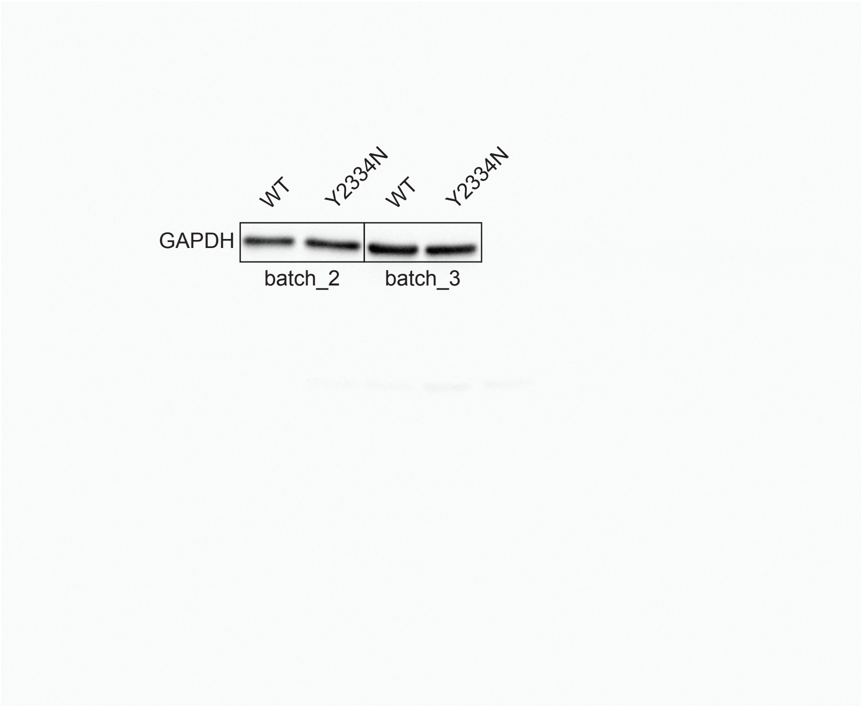

Original data for figure 1d, recoverin

The rectangle marks the analyzed data represented by the data point in figure 1d

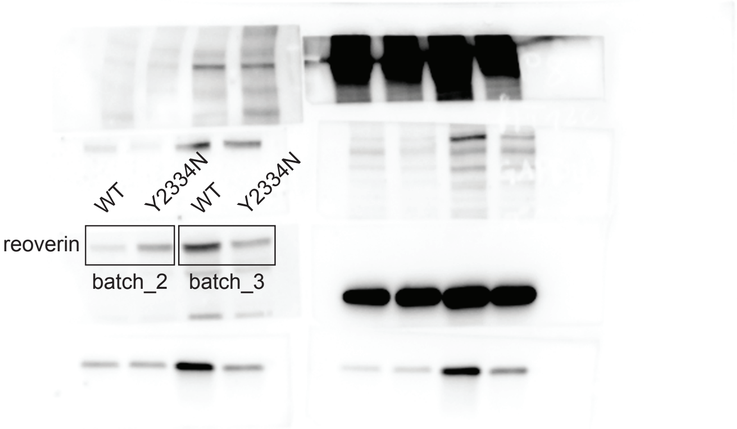

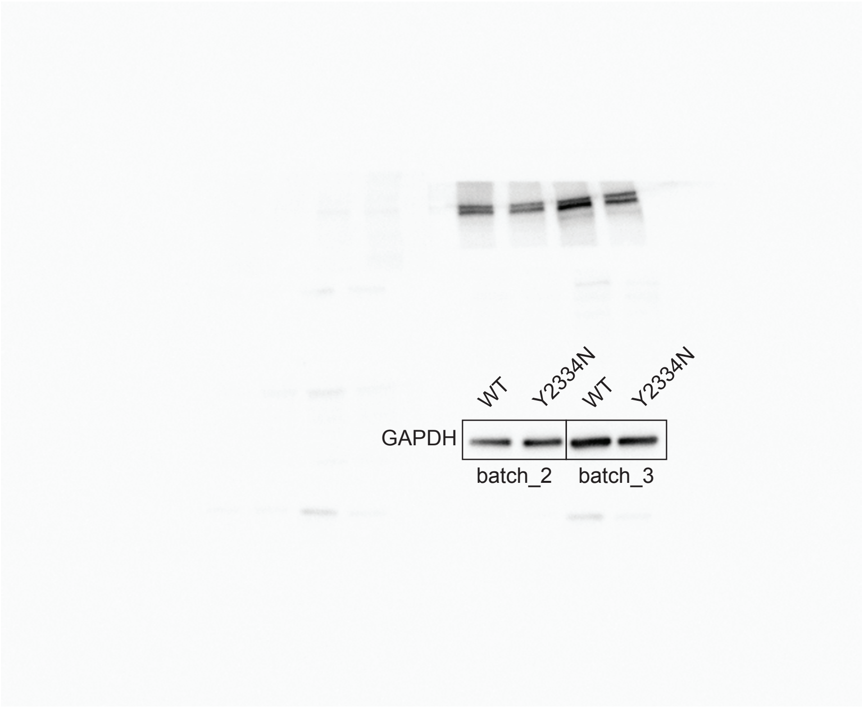

Original data for figure 1d, rhodopsin_batch2

The rectangle marks the analyzed data represented by the data point in figure 1d

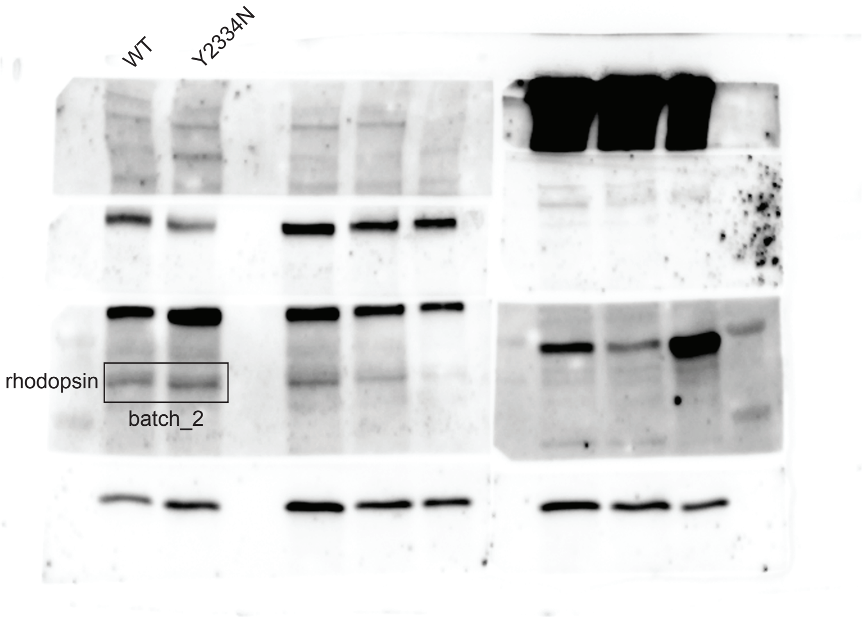

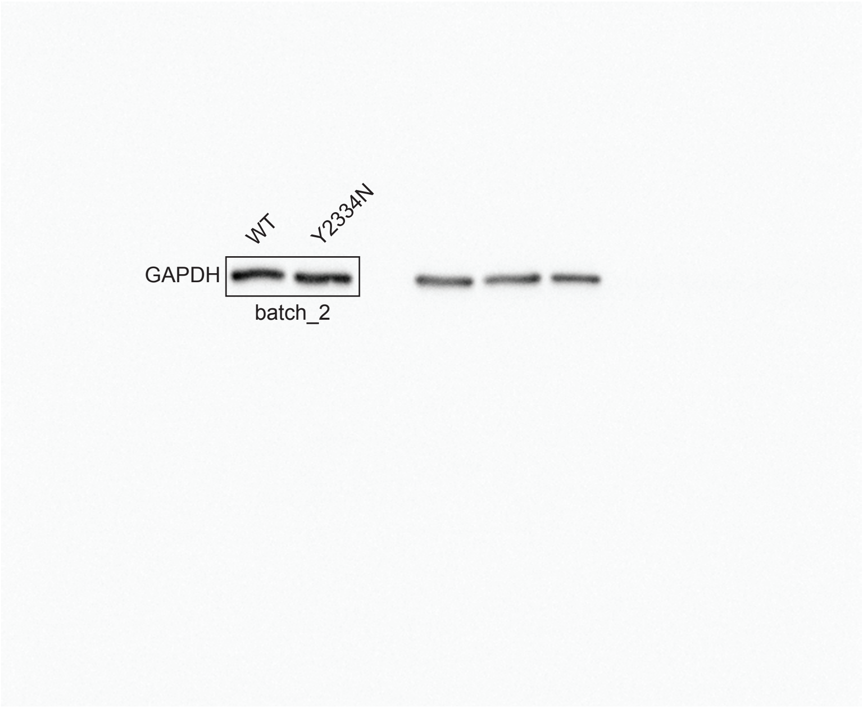

Original data for Figure 1d, rhodopsin_batch3

The rectangle marks the analyzed data represented by the data point in Figure 1d

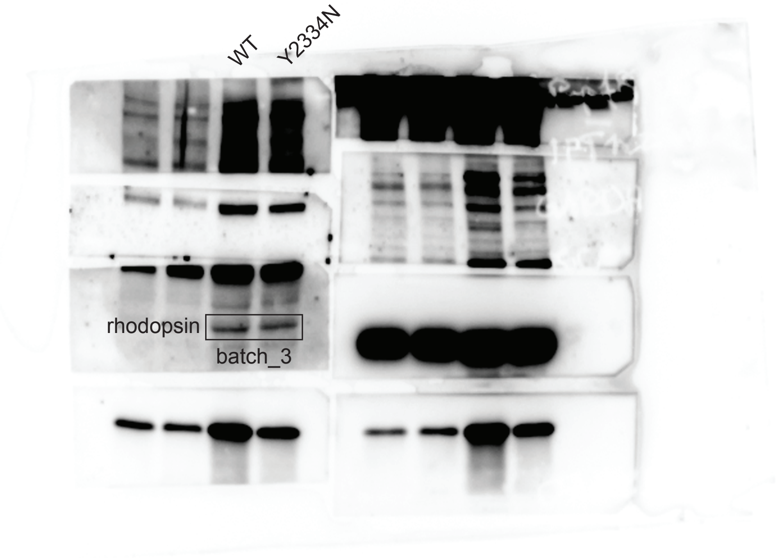

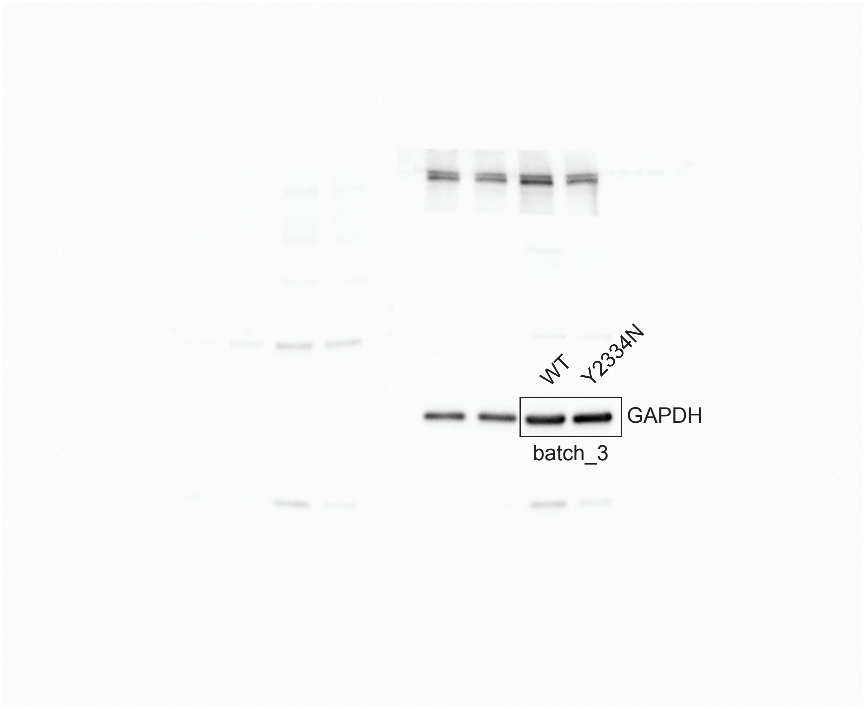

Original data for figure 1d, ROM1

The rectangle marks the analyzed data represented by the data point in figure 1d

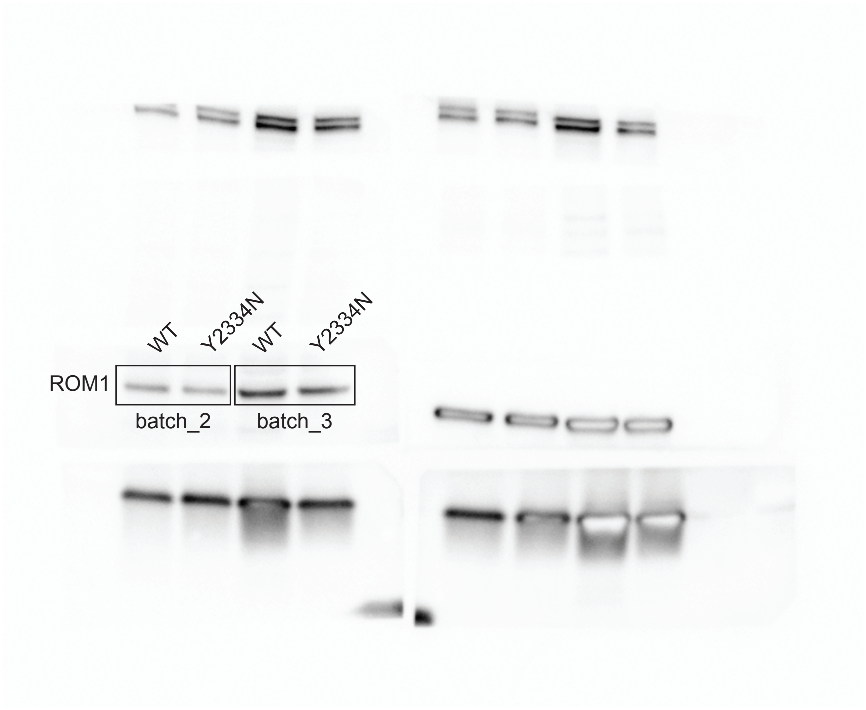

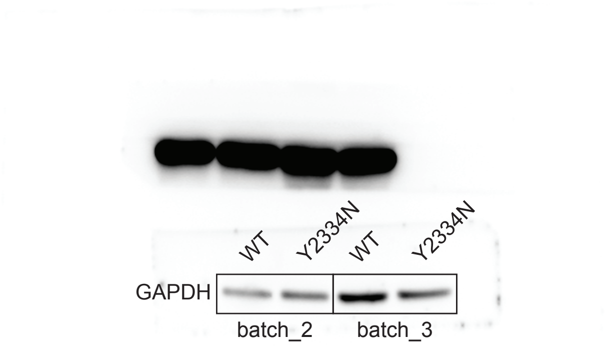

Original data for figure 1d, visual arrestin

The rectangle marks the analyzed data represented by the data point in figure 1d

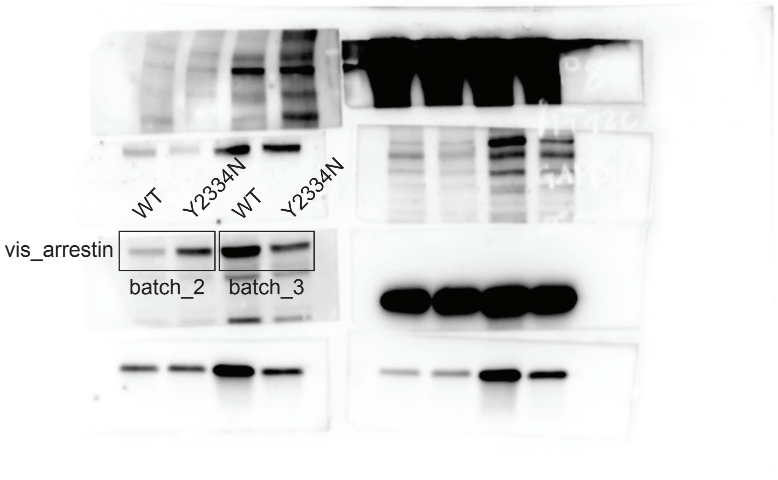

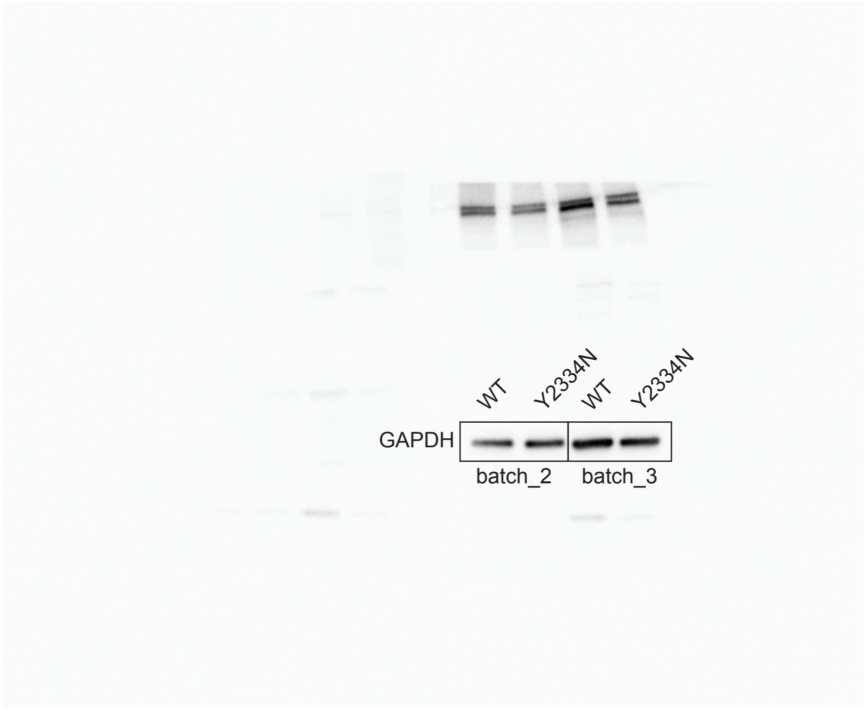

Original data for figure S1b

The rectangle marks the cropped area displayed in figure S1b

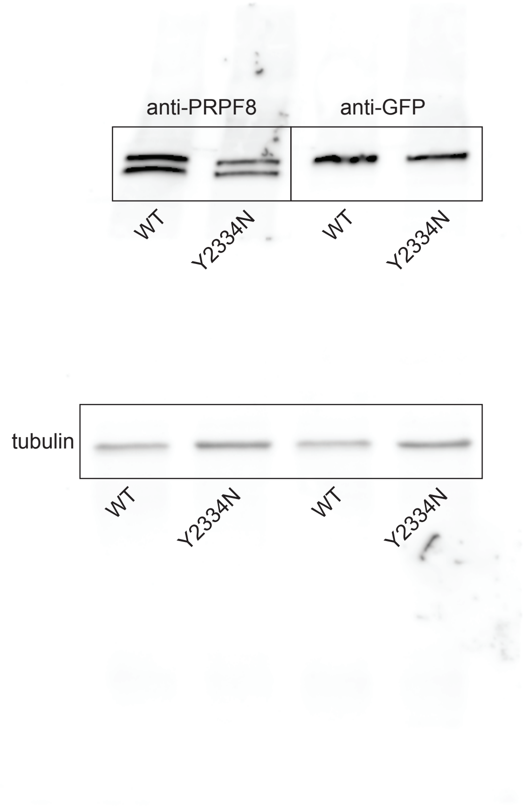

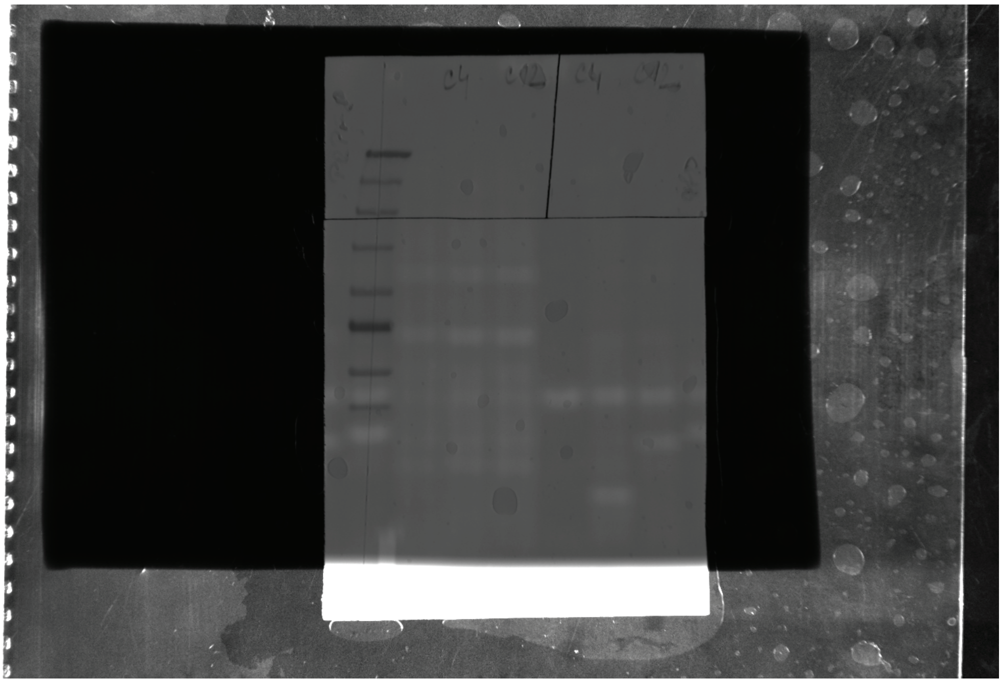

Original data for figure S1d

The rectangle marks the cropped area displayed in figure S1d

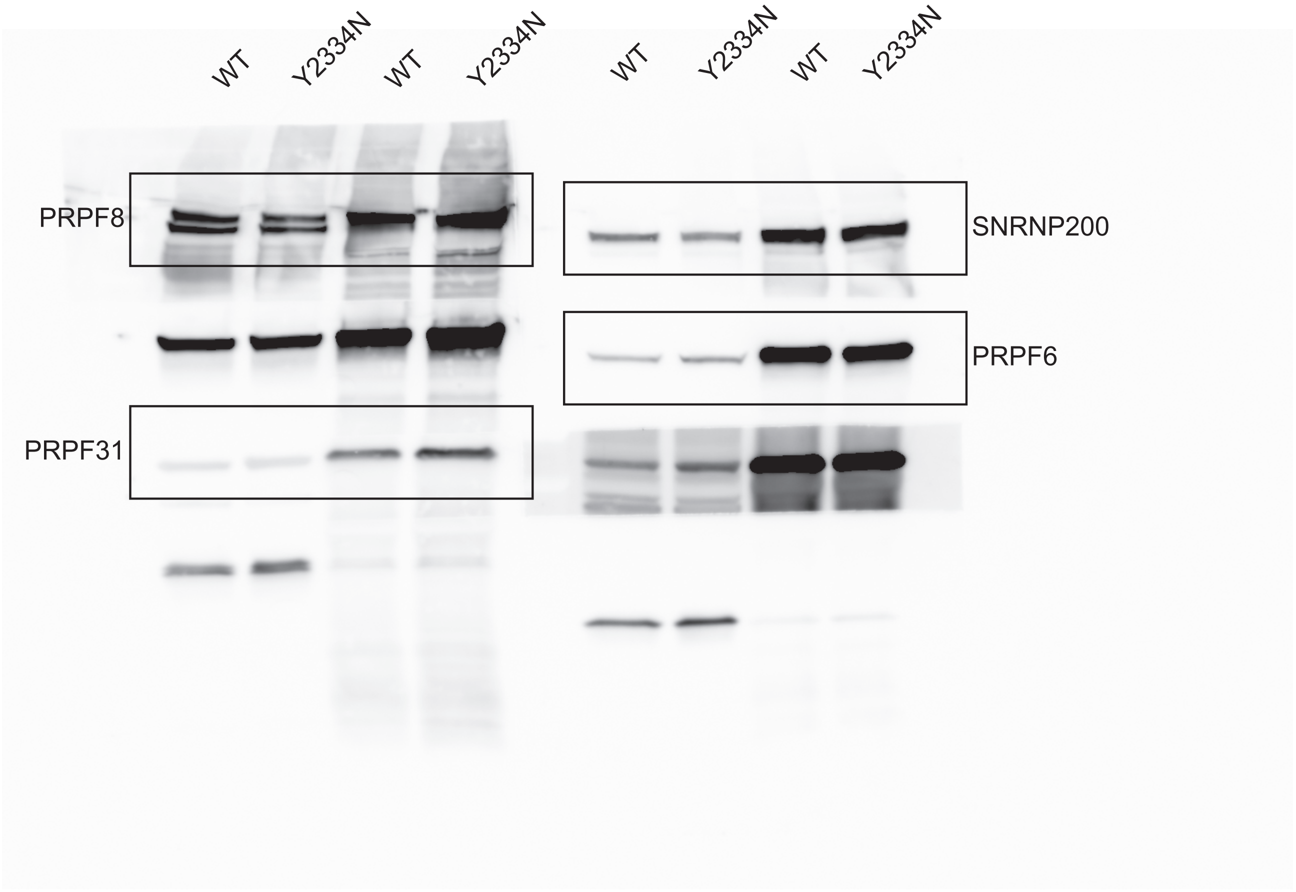

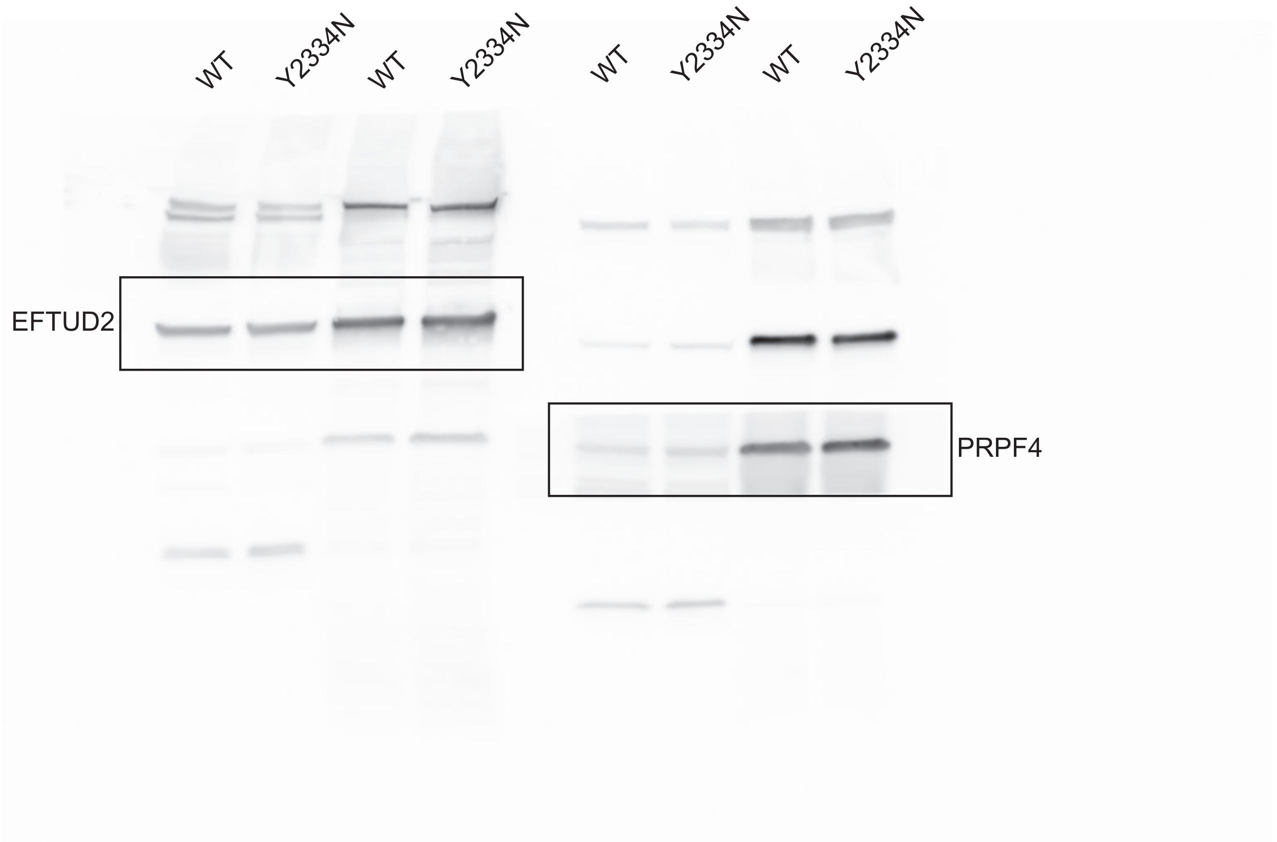

Original data for figure 1e, panel_1 (top)

The rectangle marks the cropped area displayed in figure S1e

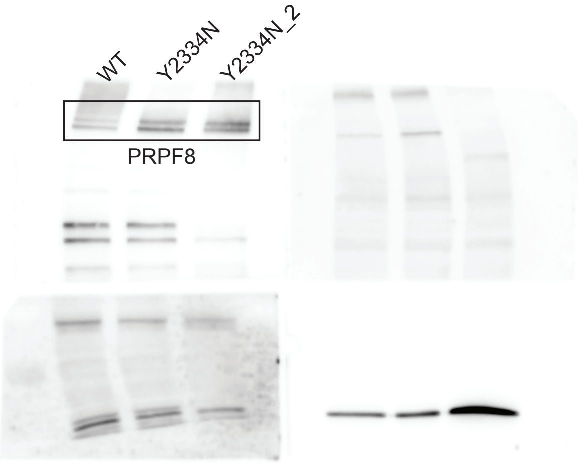

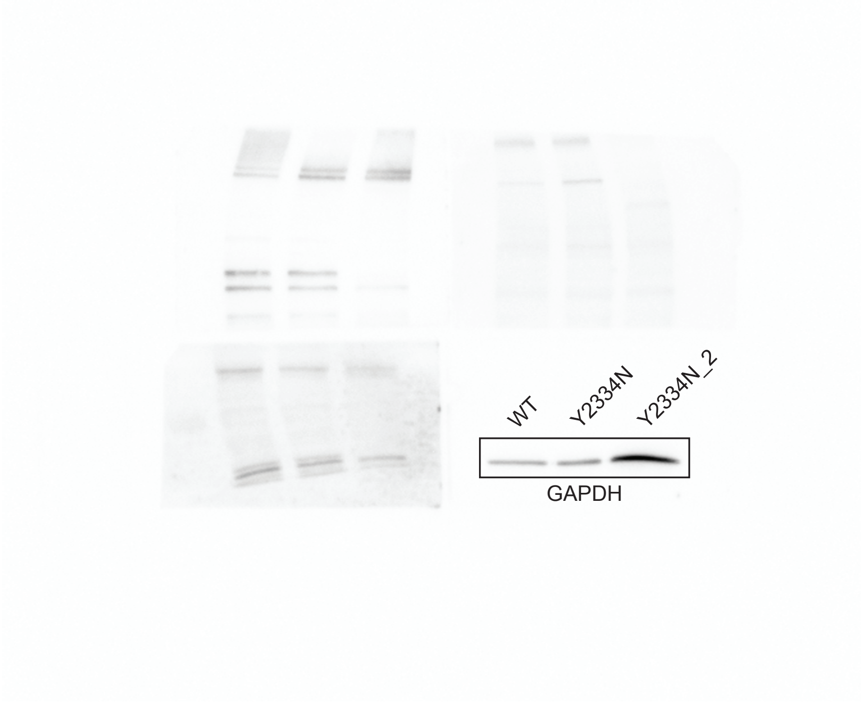

Original data for Figure 1e, panel_2 (middle)

The rectangle marks the cropped area displayed in Figure S1e

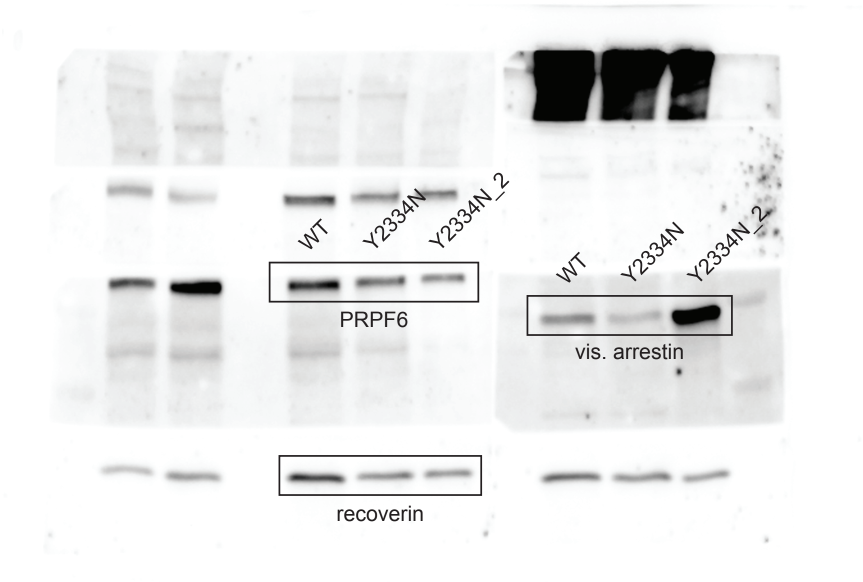

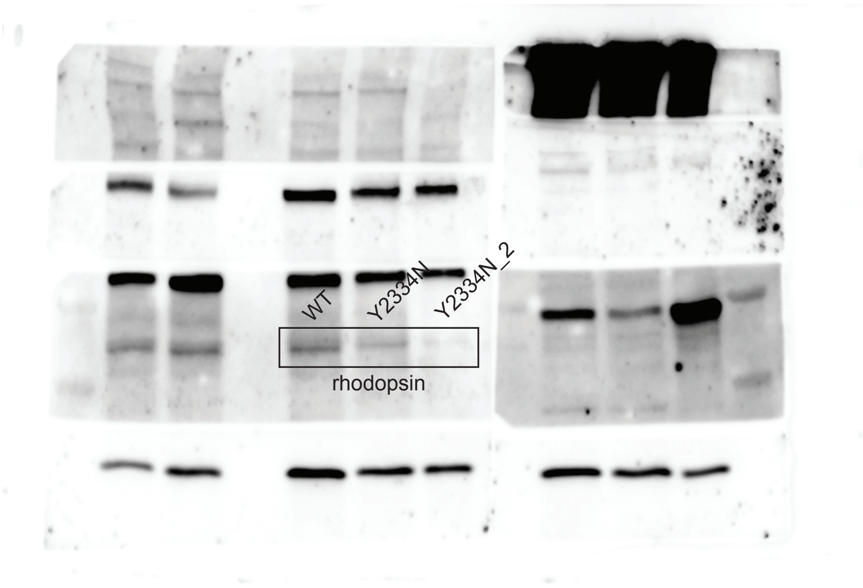

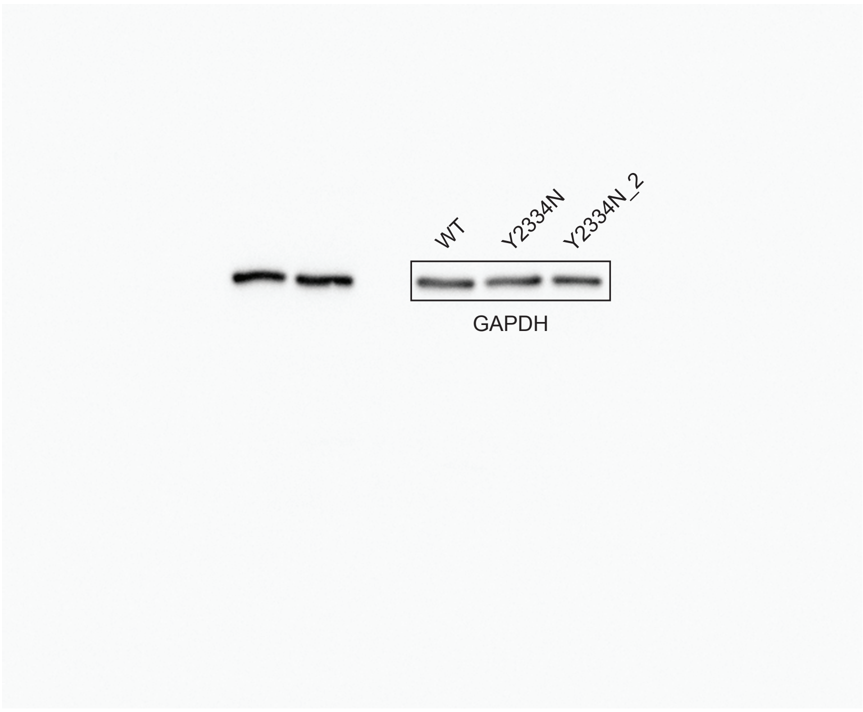

Original data for Figure 1e, panel_3 (middle)

The rectangle marks the cropped area displayed in Figure S1e

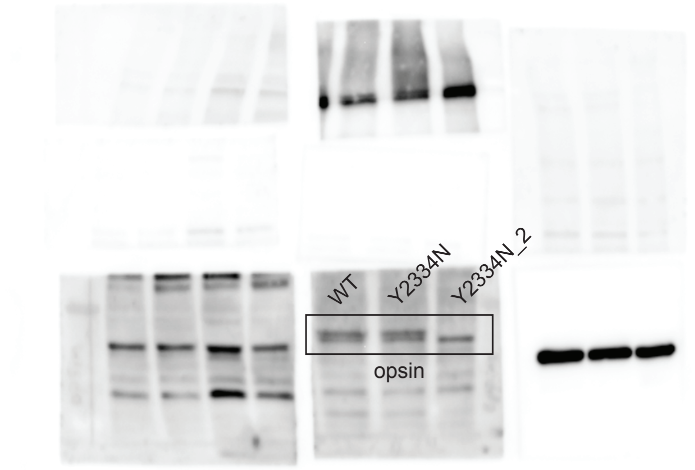

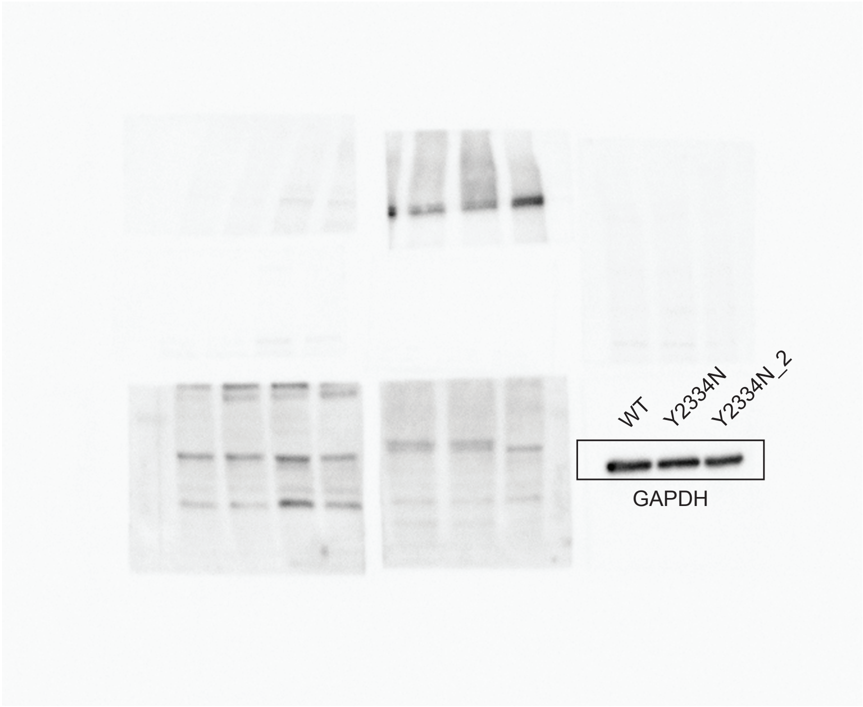

Original data for Figure 1e, panel_4 (bottom)

The rectangle marks the cropped area displayed in Figure S1e

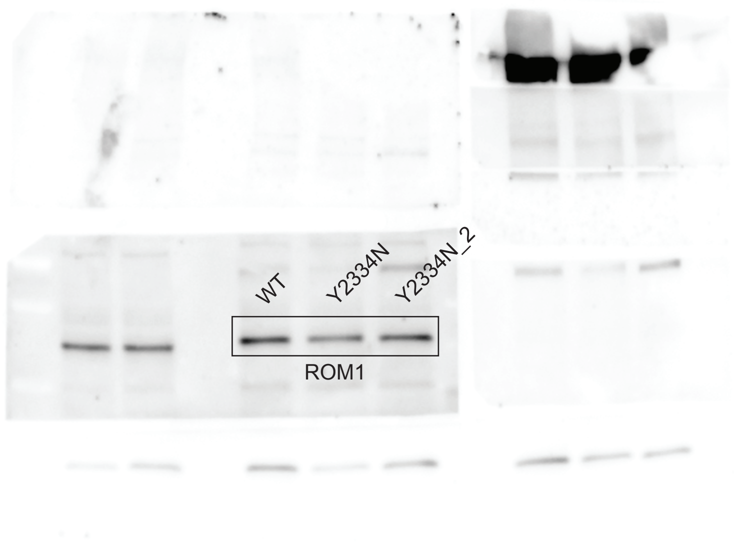

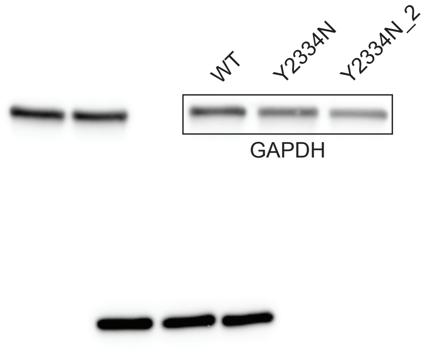

Supplement: Supplementary file 2 — Supplementary Information 2. [file 41598_2026_40376_MOESM2_ESM.pdf]
